# Death Adder Envenoming Causes Neurotoxicity Not Reversed by Antivenom - Australian Snakebite Project (ASP-16)

**DOI:** 10.1371/journal.pntd.0001841

**Published:** 2012-09-27

**Authors:** Christopher I. Johnston, Margaret A. O'Leary, Simon G. A. Brown, Bart J. Currie, Lambros Halkidis, Richard Whitaker, Benjamin Close, Geoffrey K. Isbister, for the ASP investigators

**Affiliations:** 1 School of Medicine Sydney, University of Notre Dame Australia, Darlinghurst, New South Wales, Australia; 2 NSW Poisons Information Centre, Sydney Children's Hospital Network, Sydney, New South Wales, Australia; 3 Department of Clinical Toxicology and Pharmacology, Calvary Mater Newcastle and the Discipline of Clinical Pharmacology, University of Newcastle, Newcastle, New South Wales, Australia; 4 Centre for Clinical Research in Emergency Medicine, Western Australian Institute for Medical Research, Royal Perth Hospital and University of Western Australia, Perth, Western Australia, Australia; 5 Menzies School of Health Research and Northern Territory Clinical School, Royal Darwin Hospital, Darwin, Northern Territory, Australia; 6 Emergency Department, Cairns Base Hospital, Cairns, Queensland, Australia; 7 Emergency Department, The Townsville Hospital, Townsville, Queensland, Australia; University of Kelaniya, Sri Lanka

## Abstract

**Background:**

Death adders (*Acanthophis spp*) are found in Australia, Papua New Guinea and parts of eastern Indonesia. This study aimed to investigate the clinical syndrome of death adder envenoming and response to antivenom treatment.

**Methodology/Principal Findings:**

Definite death adder bites were recruited from the Australian Snakebite Project (ASP) as defined by expert identification or detection of death adder venom in blood. Clinical effects and laboratory results were collected prospectively, including the time course of neurotoxicity and response to treatment. Enzyme immunoassay was used to measure venom concentrations. Twenty nine patients had definite death adder bites; median age 45 yr (5–74 yr); 25 were male. Envenoming occurred in 14 patients. Two further patients had allergic reactions without envenoming, both snake handlers with previous death adder bites. Of 14 envenomed patients, 12 developed neurotoxicity characterised by ptosis (12), diplopia (9), bulbar weakness (7), intercostal muscle weakness (2) and limb weakness (2). Intubation and mechanical ventilation were required for two patients for 17 and 83 hours. The median time to onset of neurotoxicity was 4 hours (0.5–15.5 hr). One patient bitten by a northern death adder developed myotoxicity and one patient only developed systemic symptoms without neurotoxicity. No patient developed venom induced consumption coagulopathy. Antivenom was administered to 13 patients, all receiving one vial initially. The median time for resolution of neurotoxicity post-antivenom was 21 hours (5–168). The median peak venom concentration in 13 envenomed patients with blood samples was 22 ng/mL (4.4–245 ng/mL). In eight patients where post-antivenom bloods were available, no venom was detected after one vial of antivenom.

**Conclusions/Significance:**

Death adder envenoming is characterised by neurotoxicity, which is mild in most cases. One vial of death adder antivenom was sufficient to bind all circulating venom. The persistent neurological effects despite antivenom, suggests that neurotoxicity is not reversed by antivenom.

## Author Summary

Death adders are a genus of venomous snakes found in Australia, Papua New Guinea and Indonesia. Death adder envenoming is a rare but important health problem in Australasia. Definite death adder bites were recruited as part of the Australian Snakebite Project (ASP). Clinical effects, laboratory results and response to antivenom treatment were recorded for each case. Death adder envenoming was confirmed by enzyme immunoassay in blood collected from patients. The most important clinical effect was neurotoxicity, which was mild in most cases. One vial of antivenom was shown to be effective at binding circulating death adder venom. However, antivenom had little effect on the neurotoxicity that developed in envenomed patients and neurotoxicity took on average one day to resolve. This study supports the idea of presynaptic neurotoxicity in death adder envenoming which was previously thought to be due to post-synaptic neurotoxicity. The study calls into question the benefit of antivenom, with poor response shown in patients with both mild and severe envenoming.

## Introduction

Death adders (genus *Acanthophis*) are a member of the snake family Elapidae. They inhabit most of Australia (excluding Victoria and Tasmania), Papua New Guinea, Irian Jaya and the Indonesian islands of Seram, Halmahera, Obi and Tanimbar. Considerable debate about the taxonomy of individual species continues, with up to 12 species of death adder being proposed [1], [2]. Death adders are readily distinguishable from other Australian snakes by their “viper-like” appearance. They also differ to other Australian elapids because they are ambush predators, being primarily active at night.

Death adder venom has been the subject of considerable *in vitro* investigation and several venom components have been isolated and identified. Until recently, post-synaptic neurotoxins were thought to be the most important components of death adder venom and numerous post-synaptic toxins have been isolated [2]–[6]. However, recent work has identified a number of pre-synaptic neurotoxins present in at least three species of death adder [7]–[9]. Several other components have been identified in death adder venom, including myotoxins, pro-coagulants, anticoagulants and toxins which interfere with platelet aggregation [10]–[17]. The clinical significance of several of these components remains unclear.

There is limited published information about the clinical effects of death adder envenoming and its treatment. Neurotoxicity appears to be the most important clinical effect of death adder envenoming [2], [18], [19]. Most of the available clinical information comes from two case series from Port Moresby General Hospital in Papua New Guinea. Campbell's 1966 paper provided the first case series of death adder bites with 15 cases [18]. It included five patients with evidence of neurotoxicity, two of whom had extensive paralysis. The study describes the successful reversal of envenoming with antivenom therapy but does not provide objective evidence of the duration of effects after treatment. Definite identification of the involved snake was only available for some of the cases. Lalloo et al reported a further 32 cases of death adder bites from Papua New Guinea with confirmation by enzyme immunoassay [19]. Neurotoxicity was a feature in 17 (53%) of these patients, with five patients requiring intubation and ventilation. The study found a variable response to antivenom, with some cases responding rapidly with a reversal of effects, and other cases not responding at all.

The limited information on Australian death adder bites does not provide a complete description of the envenoming syndrome. In a prospective series of 21 cases from tropical northern Australia, eight (38%) developed neurotoxicity [20]. There is controversy regarding the dose and effectiveness of antivenom and whether adjunctive therapy with anticholinesterases such as neostigmine play a role in treatment [2], [20]–[27]. The aim of this study is to describe the clinical syndrome of death adder envenoming in Australia and evaluate the response of death adder envenoming to antivenom therapy.

## Methods

The study is a prospective cohort study of definite death adder (*Acanthophis spp*) bites recruited to the Australian Snakebite Project (ASP).

### Materials

*Acanthophis antarcticus* (Common Death Adder) venom was purchased from Venom Supplies, South Australia. Death adder antivenom was manufactured by CSL Ltd. Polyclonal monovalent rabbit IgG to *A. antarcticus* venom was purchased from the Western Australian Institute for Medical Research. Tetramethylbenzidine (TMB) was purchased from Sigma. Rabbit IgG was biotinylated using EZ-link sulfo-NHS-LC-Biotin (Pierce #21335). Streptavidin-conjugated horseradish peroxidise was purchased from Millipore Chemicon. Skim milk ‘Diploma’ brand instant powder was prepared as a 1% solution in phosphate buffered saline (PBS) for blocking solution. Bovine Serum Albumin (BSA) was obtained from Bovostar. Greiner microlon high binding 96 well plates were used for the enzyme immunoassays. The plates were read on a BioTek ELx808 at 450 nm.

### Patients and Patient samples

Patients with death adder bites were recruited as part of the Australian Snakebite Project (ASP). ASP is an ongoing prospective multicentre study which recruits patients from over 100 hospitals in Australia. The inclusion criterion for ASP is any patient who has been bitten by a snake, either suspected or confirmed. The only exclusion criterion is any patient less than two years of age. Patients are recruited to the ASP by local investigators present in treating or referral hospitals, or by clinical toxicologists when contact is made through the Poisons Information Centre Network in Australia. Patient information, patient consent, study procedures and datasheets are faxed to local investigators and are included as Protocol S1. Ethics approval has been obtained from the human research ethics committee of the Menzies School of Health Research and 20 human research ethics committees relevant to all institutions involved in the study. Informed written consent was obtained from all patients in the study.

Information on patient demographics, bite circumstances, clinical evaluation of the patient, laboratory results, treatments given and response to therapy are recorded for all patients recruited on datasheets made available to treating doctors. All decisions about treatment of the patient are made by the treating doctor or from advice given by the National Poison Information Centre Network. This information is entered in the study database and coded to a clinical envenoming syndrome according to previously defined criteria [28].

Multiple serum samples are taken from each patient both pre- and post-antivenom administration which are then spun, aliquotted and stored at −80°C for later analysis of venom and antivenom. The pre-antivenom samples are used to confirm the presence and concentration of specific venoms. Post-antivenom samples are used to confirm the absence of detectable free venom after antivenom administration.

The ASP database was searched from January 2002 to January 2012 for all potential cases of death adder bites. Potential death adder bite cases were defined by either a positive CSL snake venom detection kit (sVDK) result, expert identification of the snake or by clinical findings that were suggestive of envenoming by death adder (eg. neurotoxicity in the absence of coagulopathy). Blood samples from these possible cases were analysed to confirm the type of venom with enzyme immunoassay if pre-antivenom blood was available. Definite cases were defined as those with positive expert identification or detection of death adder venom with specific venom enzyme immunoassay.

### Enzyme Immunoassay

Specific venom enzyme immunoassay was performed on all patient samples using a previously described technique developed for Australian snakebites [29]. All washings were done with 0.02% Tween 20 in PBS. Plates were coated with 100 µL of rabbit anti-*A.antarcticus* IgG 1 µg/mL in carbonate buffer (50 mM pH 9.6). After one hour at room temperature they were refrigerated at 4°C overnight. Each plate was washed and then blocked with 300 µL of blocking solution (dilute skim milk) to occupy free binding sites on the plates. Each plate was then washed again after one hour before adding 100 µL of patient sample, control or spiked sample to each well, as a dilution of 10% or 1% or both, in PBS. Following three washes, 100 µL of biotinylated rabbit anti-*A.antarcticus* IgG (0.2 ug/mL in 0.5% BSA/PBS) was added to each sample. After a further hour the plates were washed another three times, and 100 µL of Strepavidin-conjugated horseradish peroxidase (0.15 µg/mL in 0.5% BSA/PBS) was added. Then, after 1 hour the plates were again washed three times. Tetramethylbenzidine (100 µL) was added and the colour left to develop for two minutes. Then to each sample 50 µL of 1M sulfuric acid was added to halt the reaction. Samples are all performed in triplicate and the mean value calculated. The coefficient of variation was less than 5% for all experiments.

### Level of detection/level of blank determination

The EP17-A protocol was used to determine the limit of blank and limit of detection of the assay [30]. Previous studies of taipan (*Oxyuranus* sp.) venom have shown that using this approach the level of detection can be set as low as 0.15 ng/mL [29]. Twenty four non-envenomed serum samples were measured unspiked (blank) and spiked at a concentration such that the overlap of absorbance with the unspiked samples was ≤5%. This level was determined as 0.2 ng/ml.

### Data Analysis

Standard curves were fitted by linear and non-linear regression and the data was plotted to fit a sigmoidal dose–response curve with an r^2^ value of 0.99. Normality of the data was assessed by the Kolmogorov-Smirnov test and the Shapiro-Wilk normality test. Descriptive data is presented as medians with interquartile ranges (IQR) and ranges. All analyses and graphics were done in GraphPad Prism version 5.03 for Windows, GraphPad Software, San Diego California USA, www.graphpad.com.

## Results

From 35 potential cases identified, six were excluded because they did not have expert identification of the snake or venom detected by enzyme immunoassay. The 29 definite death adder bites are summarised in Table 1.

**Table 1:** Characteristics and demographic features of 29 patients with definite death adder bites.

**Age [yr]; median (IQR, Range)**	45 (33–52; 5–74)	
**Sex (Male)**	25	86%
**State/Territory**		
Northern Territory	8	28%
Queensland	8	28%
New South Wales	6	21%
Western Australia	4	14%
Victoria	3	10%
**Bite site** 1		
Upper Limb	20	69%
Lower Limb	8	28%
**Circumstances of bite** 1		
Intentionally interfering with snake	17	59%
Stepped on/near snake	7	24%
Clearing debris/activity near snake	4	14%
**PBI in place on arrival**	22	76%
**Antivenom given**	13	45%
**Snake Handler**	15	52%

PBI – pressure bandage with immobilisation.

1 In one non-envenomed patient no bite site or circumstances of bite were recorded.

### Demographic and bite circumstance data

The median age of the patients was 45 years (IQR: 33–52 y; Range 5–74 y,); 25 patients were male (86%). The bite site was the upper limb in 20 cases (69%), lower limb in eight cases (28%) and not known in one case. The activity that led to the bite was intentionally interfering with the snake in 17 cases (59%), stepping on or near the snake in seven cases (24%), removing debris or interfering with something on the ground near the snake in four cases (14%) and not known in one case. Bites to snake handlers made up 15 of the cases (52%). Adequate pressure bandage with immobilisation was used as first aid in 22 cases (76%). Of the 14 death adder bites that occurred in the wild, nine of them occurred in the evening/night between 6pm and 6am.

**Figure 1:**
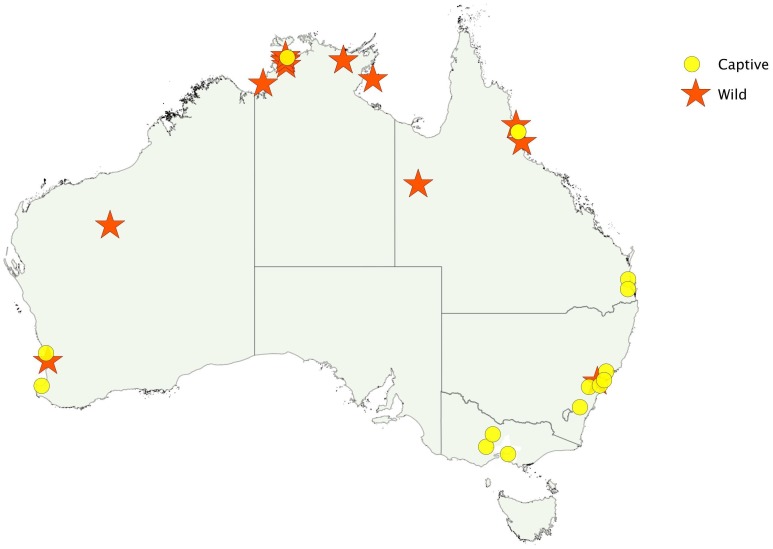
Geographical location of death adder bites, including bites by captive snakes (red star) and bites by snakes in the wild (yellow circle).

The snake was identified as a death adder by an expert in 19 cases. In nine of these the species of death adder was determined: four *A. praelongus* (Northern Death Adder), two *A. antarcticus* (Common Death Adder), two *A. hawkei* (Barkly Tableland Death Adder) and one *A. rugosus* (Rough-scaled Death Adder). There were too few envenomed cases by each species to allow any comparison between species. Bites occurred all around Australia, but bites in the wild occurred mainly in the north (Figure 1).

### Clinical Effects

**Figure 2:**
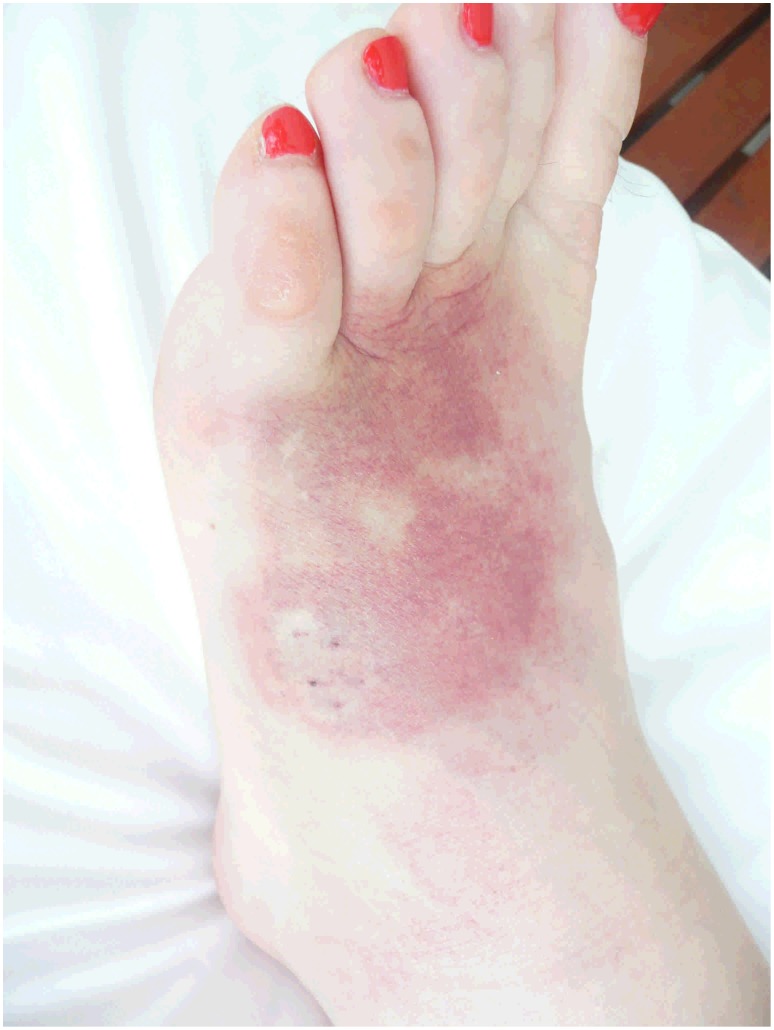
Local effects from a death adder bite to the foot.

Local and regional bite site effects occurred in 20 of the 29 patients which was most commonly pain at the bite site, but in 13 patients there were more severe effects with swelling (11) and bruising (4) (Figure 2). Over a period of days four patients went on to develop cellulitis at the bite site requiring antibiotic therapy. One of these patients required surgery for flexor sheath synovitis and one required a 48 hour intensive care unit admission for septic shock following cellulitis at the bite site.

Fourteen of the definite death adder bites resulted in systemic envenoming (Table 2). Neurotoxicity was the most common clinical syndrome, with myotoxicity in one patient and no patients with VICC. In addition to envenoming, two patients had allergic reactions to venom. There were no deaths. All patients with systemic envenoming developed local effects except two where there was no documentation of local effects.

**Table 2:** Clinical effects in 14 patients with death adder envenoming.

Clinical Effects	Number
**Neurotoxicity**	**12**
Ptosis	12
External ophthalmoplegia/diplopia	9
Bulbar weakness	7
Intercostal weakness	2
Limb weakness	2
**Local/regional bite site effects**	**12**
Bite site pain	7
Bite site swelling	7
Regional lymph node pain	7
Bite site bruising	3
**Myotoxicity**	**1**
**Non-specific systemic effects**	**3**

Neurotoxicity developed in 12 patients. In ten patients neurotoxicity was mild with paralysis limited to muscles innervated by the cranial nerves and not requiring intubation - ptosis, external ophthalmoplegia and/or diplopia, and bulbar weakness. The most common earliest indicator of neurotoxicity was ptosis in 10 patients and then ophthalmoplegia/diplopia in six. The only patient where the onset of neurotoxicity was observed more than 12 hours post bite at 15.5 hours was a five year old boy who was not woken overnight to test for ptosis. Severe neurotoxicity occurred in two patients who required intubation and mechanical ventilation for 17 and 83 hours. Table 3 provides the time of onset and resolution of neurotoxicity.

**Table 3:** Summary of patients with neurotoxicity including the time of onset and resolution and the time of antivenom.

Age/Sex	Features of Neurotoxicity^1^	Time to onset	Time to antivenom	Time to resolution
52/M	P,D,B,I,L	0.5	3.25	62
50/M	P,D,B,I,L	1	4.25	173
74/M	P,D,B	1.5	13	44.5
52/F	P	3	4.5	9.5
53/F	P,D,B	3.5	8	16.5
54/F	P,D,B	4	6.75	26
7/M	P,B	4	17.75	23
41/M	P,D	5	5.25	32.8
9/M	P,D	5.5	42	47
65/M	P,D	9.25	14	37
26/M	P,D,B	12	12.75	40
5/M	P	15.5	18.5	23.5
		4.0 hr (IQR: 2.6–6.4 hr)	10 hr (IQR: 5–15 hr)	35 hr (IQR: 24 to 45 hr)

1 P - Ptosis, D – Diplopia/External Ophthalmoplegia, B - Bulbar Weakness, I – Intercostal Weakness, L - Limb weakness.

Non-specific systemic symptoms occurred in three patients, one in which neurotoxicity did not develop. The commonest systemic symptoms were nausea and vomiting. Myotoxicity occurred in one patient bitten by a northern death adder (*A. praelongus*) who developed severe local myalgia with a maximum CK of 4770 U/L [31]

Two patients who had previously been bitten by death adders developed allergic reactions to venom without evidence of envenoming. The first patient had shortness of breath, erythema and itch to the affected arm. The second developed widespread rash, diaphoresis, urinary and faecal incontinence, and tongue/peri-orbital oedema.

### Laboratory investigations

Laboratory investigation results were available for 12 of the 14 systemically envenomed patients. All 12 patients an elevated white cell count, with a median peak value of 17.5×10^9^ (Range 11.4–41.9×10^9^) and an elevated neutrophil count, with a median value of 15.5×10^9^ (range 10.4–39.3×10^9^). Lymphocyte count was decreased in all 12 patients with a median nadir value of 0.9×10^9^ (Range 0.3–1.3×10^9^). The activated partial thromboplastin time (aPTT) was elevated in two envenomed patients (45 and 49 sec) who had normal fibrinogen levels. A bite site sVDK was performed in eight of the 14 envenomed cases, which were all positive for death adder. In a further three of the 14 a urine sVDK was done and all three were positive for death adder venom.

### Treatment

Antivenom was administered to all 12 patients with evidence of neurotoxicity and to the patient with myotoxicity. All patients received CSL monovalent death adder antivenom and an initial dose of one vial of antivenom. Subsequent doses of antivenom were administered to eight of the patients. The median dose of antivenom administered was 2 vials (Range 1–5) and the median time from bite to antivenom was 10 hours (IQR: 5–15 hr).

The median time of resolution of all features of neurotoxicity after antivenom therapy was 21 hours (IQR 5.0–28.5 hr; Range 5–168 hr). The early use of antivenom did not appear to be associated with less severe neurotoxicity. In the two patients with increased aPTT results, the aPTT returned to the normal range rapidly after these patients received antivenom.

Systemic hypersensitivity reactions occurred in five patients receiving antivenom. These were mild (limited to the skin) in four cases however one anaphylactic reaction occurred with urticaria, wheeze, dyspnoea and chest tightness. Antivenom was not stopped in any patients because of these reactions.

Neostigmine was administered to four patients including the two patients with severe neurotoxicity requiring intubation. In the first intubated patient, a dose of 2.5 mg of neostigmine was given with 600 mcg of atropine after intubation. Although the ptosis appeared to resolve the patient had ongoing respiratory weakness requiring intubation for a further five hours. The second intubated patient received three boluses of 0.5 mg, 2.5 mg and 2.5 mg of neostigmine followed by an infusion of 20 mcg per hour for 24 hours. After an initial improvement with eye opening, no further improvement in neurotoxicity was observed and the infusion was ceased due to bradycardia. Two other patients received neostigmine and atropine therapy with no demonstrated clinical benefit.

### Venom immunoassays

Pre-antivenom blood samples were available for 10 of the 12 patients with neurotoxicity and the one patient who had non-specific systemic effects in isolation. The median peak venom concentration was 22 ng/mL (IQR: 8.5–29 ng/mL, range 4.4–245 ng/mL). The patient who developed myotoxicity had no detectable venom in his pre-antivenom blood sample but that sample had measurable human IgG against death adder venom, consistent with his previous death adder bite. Death adder venom was detected in blood samples from four patients who did not have evidence of systemic envenoming, with concentrations of 0.7, 0.9, 3.7 and 40 ng/mL.

In the eight cases where post-antivenom blood samples were available, including the patient with the highest venom level of 245 ng/mL, there was no detectable venom. All of these patients had only received their first vial of death adder antivenom before this blood sample was taken.

## Discussion

This study confirms that neurotoxicity is the main feature of envenoming by Australian death adders and that in the majority of cases severe life-threatening paralysis does not develop. Measurement of venom concentrations demonstrated that all free venom was bound after one vial of death adder antivenom so larger doses are not required. This included patients envenomed by four different death adder species. Neurotoxicity did not appear to resolve or reverse with the use of antivenom. Previously reported possible effectiveness of anticholinesterates [27], [32]–[34] was not supported by this study, with the use of neostigmine providing little benefit in four cases. Given that neurotoxicity did not appear to resolve or reverse with the use of antivenom or anticholinesterases it is most likely to be due to irreversible injury from presynaptic neurotoxins, similar to the neurotoxicity from other Australian elapids such as the taipan [2], [35]. Although it is possible that antivenom prevented those with mild neurotoxicity from getting progressively worse, this only occurred in one patient (Table 3, Row 4) and the two patients with severe neurotoxicity received antivenom within 6 hours (Table 3).

The current recommended guidelines for snake bite patients in Australia of observation for at least 12 hours and not discharging at night, are well supported by the data in the study. Only one patient had delayed onset of neurotoxicity (15.5 hours), and it is likely that earlier onset was not picked up due to limited observations overnight, particularly waking this child to assess ptosis. A previous analysis of ASP data showed that all cases of neurotoxicity developed within 12 hours [36].

Just over half of death adder bites were in snake handlers which is the reason that a number of bites occurred in regions where death adders do not occur in the wild, such as Victoria. It also explains, in part, the reason for the predominance of upper limb bites, when compared to other studies of Australian snakes [37]. The study supports the fact that death adder bites from snakes in the wild are rare. In addition, they are more likely to occur at night and when the snake is disturbed. Unlike other Australasian elapids death adders are ambush predators and they are most active nocturnally.

In this study it has been shown that patients bitten by death adders have a characteristic pattern of white blood cell count changes, namely leucocytosis, neutrophilia and lymphopenia. This has been previously reported for snake envenoming [38], but because it is less reliable in some snake species such as brown snakes [36], it is only helpful if the snake is known to be a death adder. An anticoagulant type coagulopathy was seen in two patients, an effect that has not been described in Australian death adder bites before. This is similar to the coagulopathy seen in Australian black snake bites with an elevation of the aPTT [37] and although a useful marker of envenoming is unlikely to be clinically significant. Normal fibrinogen levels confirm that it is not a consumption coagulopathy.

Low concentrations of death adder venom were detected in blood samples from three patients who had no signs of systemic envenoming. These three concentrations were below those of any of the patients that developed neurotoxicity, indicating that a threshold blood concentration may need to be reached before neurotoxicity occurs. There was one patient with a high death adder venom concentration in their blood sample without any evidence of envenoming, indicating possible inter-person variability in susceptibility to venom effects and/or inter-snake variability in venom toxin composition [10], [39]

Some possible limitations of the study include the method of selection of cases for examination which in some patients was based upon prior knowledge of the syndrome of death adder envenoming. This may mean that some cases of death adder bite may have been missed and may be the reason for the much higher incidence of neurotoxicity in our study compared to previous studies. Because of the rarity of death adder bites and the small number of some species of death adder included in this study, other undescribed clinical effects of venom may occur.

This study raises important questions about neurotoxicity, including the failure of antivenom to reverse neurotoxicity in death adder bites. This is consistent with the recent isolation of pre-synaptic neurotoxins in death adder venoms. It supports presynaptic neurotoxicity being the predominant cause of neurotoxicity in death adder bites as with other neurotoxic Australasian elapids. This is also consistent with a recent study that has shown that the rarity of neurotoxicity in brown snake envenoming is due to presynaptic neurotoxins in brown snake venom being less potent and only being a small proportion of the venom [40].

Our study suggests that severe neurotoxicity developed early and rapidly and that early antivenom (within six hours) in these two patients did not prevent neurotoxicity (Table 3). This differs to a previous study from Papua New Guinea which found that early antivenom treatment within 4 hours of a taipan bite was associated with a lower incidence of intubation/ventilation (33% compared to 66%) [41]. Conversely, less severe neurotoxicity had a later onset, and the use of antivenom in these patients also did not appear to change the natural course of neurotoxicity, except possibly in one patient (Table 3, Row 4).

The median time to antivenom in the study was 10 hours, which is much longer than for treating venom induced consumption coagulopathy [42]. Table 3 shows that antivenom was given in response to the development of neurotoxicity accounting for this longer time to administration, which also means it is probably being given too late. It could be argued that antivenom may have limited benefit in the treatment of death adder neurotoxicity and that antivenom should only be given early in patients prior to the rapid onset of neurotoxicity. However, larger and controlled studies are required to determine this.

Death adder envenoming is rare in Australia but can potentially cause severe neurotoxicity as seen in two of 14 envenoming cases in this study. Given the potential for severe neurotoxicity, the snake's wide distribution and the presence of the snake in private collections, it remains an important snake to consider. Whilst most cases are not life threatening, the failure of antivenom and anticholinesterases to reverse neurotoxicity once it is established is a concern. In the absence of a randomised controlled trial demonstrating the effectiveness of antivenom to prevent the development of neurotoxicity, and given the demonstrable risk of giving antivenom (systemic hypersensitivity and anaphylaxis), the potential benefit of early antivenom remains uncertain.

## Supporting Information

Protocol S1 **Trial protocol, patient information sheets and data collection sheets for the Australian Snakebite Project.**
(PDF)
