# Supplementary material for: Death Adder Envenoming Causes Neurotoxicity Not Reversed by Antivenom - Australian Snakebite Project (ASP-16)
Source: PLoS Negl Trop Dis. 2012 Sep 27;6(9):e1841. doi: 10.1371/journal.pntd.0001841 (PMC3459885; doi:10.1371/journal.pntd.0001841)
Supplement: Protocol S1 — Trial protocol, patient information sheets and data collection sheets for the Australian Snakebite Project. (PDF) [file pntd.0001841.s001.pdf]

ASP Australian Snakebite Project PROCEDURE

19-11-2008
V1- NR-Menz

AIMS (1) To investigate the appropriate use and safety of snake antivenoms in Australia.
(2) To investigate the efficacy and safety of fresh frozen plasma (FFP) for treating patients with severe venom-induced consumption coagulopathy (VICC).

Inclusion Criterion – Any patient who has been bitten by a snake, whether definite or suspected.

Exclusion Criterion – Age <2years.

PROCEDURE

Step 1 – At any time that blood is taken for routine care (FBC, COAGS/D-dimer, CUE, LFT, CK, LDH, BSL), take additional research blood; a plain/serum tube (colour varies between labs). Note on all request forms "Australian Snakebite Project".

Step 2 – Call an ASP investigator on the mobile number below. We will fax the necessary paperwork to you (if this is not already to hand) and liaise with your pathology service.

Step 3 – Obtain consent as soon as the patient's condition permits (or from next of kin) and immediately fax the completed Consent Form and Datasheet 1 to the fax number below.

Step 4 – Start filling out remaining datasheets: Keep these with the patients notes and continue recording relevant clinical data.

Step 5 – Proceed according to each coagulation study result as follows:

INR, PT or aPTT more than twice the normal upper limit and D-dimer high?

No

1. Standard care
2. Additional research bloods whenever routine bloods are taken
3. Antivenom use as indicated, e.g. for neurotoxicity

Yes

1. Repeat research blood just prior to antivenom
2. Give appropriate antivenom according to local snake species or venom detection kit (VDK) results. ***We recommend two vials as the maximum dose of brown snake antivenom.***
4. At **3 hours** post-antivenom take research blood tubes only
5. At **6 hours** and **12 hours** post-antivenom and then **every 12 hours until discharge** repeat routine bloods (including COAGS) + take research blood tubes. ***Please call us on one of the phone numbers below just prior to discharge so that we can make appropriate follow-up arrangements with the patient.***

Step 6 – Record all premedications, reactions to antivenom (AV), reactions to FFP, and bleeding complications on the study datasheet. **If a reaction to AV or FFP occurs, call the National Study Line / Investigator and take additional samples as outlined on the adverse reaction datasheet.**

Step 7 – Fax all completed Datasheets 1-4 (re-send Datasheet 1) to the fax number below.

National Study Line (24 hours): 1800 676 944 (IF THIS FAILS then contact a Chief Investigator directly: Dr Geoff Isbister **0438 466471** or A/Prof Simon Brown **0419 796678**

Fax number for submitting consent forms and datasheets: (08) 9224 1489

Australian Snakebite Project (ASP)

Guidelines for the management of anaphylaxis to antivenom

(i) Preparation prior to commencing antivenom.

a. We do <u>not</u> recommend routine premedication with antihistamines or steroids

b. Dedicate one small bore (18-20 G in adults) IV line to antivenom administration and one large bore IV line (16-14 G in adults) for emergency resuscitation.

c. Prepare 1L Normal Saline (20 ml/kg in children) ready to give under pressure.

d. Prepare adrenaline 1:1000 (1mg in 1 mL) drawn up to a dose of 0.01 mg/kg (max. 0.3 mg, i.e. max 0.3 mL) and label "adrenaline for i.m. injection only (dose in mg)".

e. Prepare an i.v. infusion of adrenaline 1mg in 100 mL (controlled by infusion pump or syringe driver) ready to attach by a side arm to the resuscitation line. Anti-reflux valves must be attached above the side arm on any other infusions using this i.v., to prevent adrenaline going back up into the other fluid bags. To prevent erroneous administration, do not attach the adrenaline infusion unless it is needed.

f. Record blood pressures on the other side to the fluid/adrenaline infusion, to avoid pronged cuff inflations and thus extravasation of infusion fluids.

(ii) Management of a reaction (In addition to <u>study procedures – see ASP Datasheet 4</u>)

a. Most reactions are related to the rate of antivenom infusion, and cause flushing, hypotension and bronchospasm. Some mild reactions resolve with temporary cessation of the antivenom infusion and recommencing it at a slower rate.

b. Envenomed patients may be severely coagulopathic, so it is important to be cautious when giving adrenaline to avoid blood pressure surges, which might lead to intracerebral haemorrhage.

c. Initial management of severe reactions (sudden hypotension, bronchospasm):

   i. Suspend the antivenom infusion.

   ii. Lie the patient flat (if not already), commence high flow/100% oxygen and support airway/ventilation as required.

   iii. Rapid infusion of 1L N Saline (20 mL/kg in children) over 2-3 minutes.

   iv. Adrenaline i.m. into the lateral thigh, 0.01 mg/kg to maximum of 0.3 mg (alternatively, those experienced with i.v. adrenaline infusions may proceed directly to this, as below).

   v. Liaise with toxicology service regarding ongoing management.

d. For reactions that do not respond to initial management:

   i. If hypotensive, repeat Normal Saline bolus as above (up to 50 mL/kg may be required).

   ii. Commence i.v. infusion of adrenaline (0.5-1 mL/kg/hour, of 1 mg in 100 mL) and titrate according to response; monitor BP every 3-5 minutes (using the arm opposite to the infusion); beware that as the reaction resolves adrenaline requirements will fall, the blood pressure will rise and the infusion rate will need to be reduced.

   iii. Consider nebulised salbutamol for bronchospasm, nebulised adrenaline for upper airway obstruction, and i.v. atropine for severe bradycardia.

   iv. Seek advice urgently from the local/regional ED Consultant &/or ICU Consultant.

REFERENCE: Snakebite and Spiderbite Management Guidelines SA. Prof. Julian White.
Government of South Australia Department of Health Guideline Ref G0034, August 2006.

Tropical Toxinology Unit
Menzies School of Health Research and
Royal Darwin Hospital

Prof Bart Currie
Assoc Prof Geoff Isbister
Assoc Prof Simon Brown
Email: Geoff.isbister@gmail.com
Mobile : 0438 466 471 / 1800 676 944

PO Box 41096 Casuarina
Northern Territory 0811, Australia

19 September 2008

Australian Snakebite Project (ASP)
Information Sheet

You are invited to take part in the *Australian Snakebite Project*, being conducted by Menzies School of Health Research. This is a research project and participation is entirely voluntary. You can withdraw from the study at any time.

Background and Aims of the Study

Spider and snake bites are not uncommon in Australia and antivenoms exists for the treatment of many of these bites. However, despite this, there are still many questions about the effects of different venoms and about the exact amount of antivenom that is required for treatment.

This study will measure the venom levels in blood after a sting or bite by a venomous animal (snake or spider). This aims to help us determine whether venom levels are important in predicting the severity of certain envenomations, and whether they correlate with the effects of the bite or sting. The study will also be able to determine how long the human body takes to excrete the toxins ie. how long the effects of the envenomation will take to wear off. Finally the study will look at the effects that treatment with antivenom has on venom levels to help establish the correct amount of antivenom to use.

Your Involvement

If you agree to take part in this study you will be required to give a number of extra blood samples while you are in hospital to measure the venom and antivenom levels in your blood. This will require the insertion of an intravenous cannula into a vein in your hand or arm at the start of the study. This will then be used to take a number of samples of blood throughout the course of the study to minimise the discomfort.

Depending on how long you need to remain in hospital, up to 4 samples of blood will be taken each day. In the majority of cases the blood will be taken at the same time as you would have blood collected for the treatment of the sting or bite.

The only risk to being involved in the study is the additional need for an intravenous cannula. This will be inserted by experienced health care staff. There are minimal risks from venepuncture, but they include a small risk of bruising at the site and the small chance of an infection developing from the presence of the cannula. The standard precautions of using a sterile technique to collect blood and insert the cannula will significantly reduce the risk of this and will be adhered during the study.

Participation in the Study

Participation in the study is completely voluntary and you will suffer no disadvantage if you elect to not be involved in the study and will continue to receive optimal ongoing care. You may withdraw from the study at any time.

If you do participate in the study you will receive exactly the same treatment as if you were not involved in the study. The only difference will be the collection of extra blood samples.

Use of the data collected

The information collected from this study will be stored in a de-identified fashion. You can be assured that all records dealing with your participation in this study will be kept under safe storage for 15 years after completion. Authorised persons within the institution may also inspect records for purposes of data audit only. Individual participants in the study will not be identifiable in any reports of the data from the protocol or any publications resulting from the research.

If you have any questions, you may contact Geoff Isbister or Bart Currie at the Menzies School of Health Research on 8922-8196 or discuss this with your treating doctors.

Any concerns, enquiries or complaints about the conduct of the study should be directed to the Secretary, Human Research Ethics Committee of the NT Department of Health and Community Services and Menzies School of Health Research, phone 8922-7922.

This form is for you to keep.

Dr Geoff Isbister

Senior Research Fellow
Menzies School of Health Research

Tropical Toxinology Unit
Menzies School of Health Research and
Royal Darwin Hospital

Prof Bart Currie
Assoc Prof Geoff Isbister
Assoc Prof Simon Brown
Email: Geoff.isbister@gmail.com
Mobile : 0438 466 471 / 1800 676 944

PO Box 41096 Casuarina
Northern Territory 0811, Australia

19 September 2008

VOLUNTEER CONSENT FOR INCLUSION IN THE

Australian Snakebite Project (ASP)

I, …………………………………………….. have been asked to participate in the above study under the direction of Dr Geoff Isbister. I understand that while the study will be under his supervision, other professional persons may assist or act on his behalf.

I have been given clear verbal information about this study and have read the attached 'Information Sheet'. I understand the general purposes and methods of the study and have been given time to consider whether I want to take part.

I have been told that there is no additional risk to being involved in this study, as the only requirement is that more blood be taken when bloods are normally taken as part of the routine care for snakebite. I have been able to ask questions and all questions have been answered satisfactorily.

I know that I do not have to take part in the study and that I can withdraw or be withdrawn by the doctor in charge at any time during the study and continue to receive appropriate treatment. My participation in the study does not affect any right to compensation, which I may have under statute or common law. I agree to the publishing of results of this study, provided my name or other identifying information is not used. I agree to be contacted by phone for follow up once I am discharged.

I consent to additional blood samples being taken and donate those specimens for the purpose of this study. In making my donation of blood, I understand and agree that the blood, and all its constituents, will be used only in relation to the above clinical research purpose. The blood, and all its constituents, may be stored to enable future testing in relation to this research project and related future research. No other researchers have access to blood samples.

I hereby voluntarily consent and offer to take part in this study.

…………………………………	…………………	…………….
Name of Volunteer	Signature of Volunteer	Date
……………………………………..	……………………..	…………….
Name of Witness to Volunteer's signature	Signature of Witness	Date
……………………………………..	……………………..	…………….
Name of treating doctor	Signature of treating doctor	Date

Tropical Toxinology Unit
Menzies School of Health Research and
Royal Darwin Hospital

Prof Bart Currie
Assoc Prof Geoff Isbister
Assoc Prof Simon Brown
Email: Geoff.isbister@gmail.com
Mobile : 0438 466 471 / 1800 676 944

PO Box 41096 Casuarina
Northern Territory 0811, Australia

19 September 2008

NEXT OF KIN, PARENT or GUARDIAN CONSENT FOR PATIENT INCLUSION IN THE *Australian Snakebite Project (ASP)*

I, ……………………………...……………….. in the capacity of 'Next of Kin' / Parent / Guardian (delete as appropriate) have been asked to give my consent for:

……………………………..……………… (patient name) to participate in the above study under the direction of Dr Geoff Isbister. I understand that while the study will be under his supervision, other professional persons may assist or act on his behalf.

I have been given clear verbal information about this study and have read the attached 'Information Sheet'. I understand the general purposes and methods of the study and have been given time to consider whether I want the above person to take part. I have been told that there is no additional risk from the patient being involved in this study, as the only requirement is that more blood be taken when bloods are normally taken as part of the routine care for snakebite. I have been able to ask questions and all questions have been answered satisfactorily.

I know that the study is voluntary and that the patient can withdraw or be withdrawn by the doctor in charge at any time during the study without affecting his/her future medical care. I agree to publishing of results of this study provided the patient's name or other identifying information is not used. I agree for the patient to be contacted by phone for follow up once I am discharged.

I consent to additional blood samples being taken from the patient. I understand and agree that the blood, and all its constituents, will be used only in relation to the above clinical research purpose. The blood, and all its constituents, may be stored to enable future testing in relation to this research and related future research. No other researchers have access to blood samples.

I hereby voluntarily consent to the patient taking part in this study.

……………………………………..	…………………	…………….
Name of Next of Kin	Signature of Next of Kin	Date
……………………………………..	……………………	…………….
Name of Witness to Next of Kin's signature	Signature of Witness	Date

<u>The treating doctor</u>
I hereby declare that I have given the Next of Kin the 'Volunteer Information Sheet' containing the information referred to above and believe he/she has understood it.

……………………………………..	………………………………	………….
Name of treating doctor	Signature of treating doctor	Date

ASP — Australian Snakebite Project — Datasheet 1

Including ASP-FFP

RESEARCHER OFFICE ONLY
STUDY ID NUMBER: ______

DOCTORS NAME ______

Patient contact telephone number(s) ______

HOSPITAL: ______

Patient Name and URN:

or Patient Sticker Label

Arrival Date: ______ (dd / mmm / yyyy) Arrival Time: __ : __ (24 hour clock)

Pt. SEX: M / F Date of Birth: ______ (dd / mmm / yyyy)

PREVIOUS HOSPITAL (If transferred): ______

HISTORY OF BITE AND FIRST AID

Bite Date: ______ (dd / mmm / yyyy) Time: __ : __ (**24 hour clock**) Part of body bitten: ______

Snake clearly seen to bite? ☐Yes ☐No Nearest suburb/town/landmark: ______

Circumstances of bite (activity at the time): ______ Number of bites: ______

Symptoms/signs so far: SEE NEXT PAGE (Clinical Datasheet 2) Time of symptom onset: __ : __ (**24 hour clock**)

Has the patient been immobilised (kept on a stretcher/not walking) since the bite? ☐Yes ☐No

Has a PRESSURE BANDAGE +/- SPLINT been applied prior to arrival at this hospital? ☐Yes ☐No

If **Yes:** Who was it first applied by? Text Time first applied: __ : __

Was it further reinforced (improved) by a health professional BEFORE arrival at this hospital? ☐Yes ☐No Time reinforced: __ : __

Was it removed BEFORE arrival at this hospital? ☐Yes ☐No Time removed? __ : __

24 hour clock

INITIAL ASSESSMENT & ACTIONS

Was a pressure bandage in place PRIOR TO arrival? ☐Yes ☐No

Characteristics:
- ☐ **Loose** &/or one layer only &/or part of limb only
- ☐ **Firm,** 2 or more layers, whole limb, well appplied
- ☐ Acting as a venous torniquet- limb swollen
- ☐ Acting as an arterial torniquet- limb ischaemic

Was the bitten limb splinted PRIOR TO arrival? ☐Yes ☐No

Splinted with: ______

Was pressure-immobilisation/splinting applied or improved on or after arrival in this hospital? ☐Yes ☐No

VDK / SNAKE ID (If available/performed)

Cut window over suspected bite site - are TEETH/FANG MARKS clearly seen? ☐Yes ☐No How many? ______

VENOM DETECTION KIT (VDK) tests performed by: ☐ LAB (Preferred) ☐ ED Doctor

VDK Result **BITE SITE**: ______

VDK Result **URINE** (Only required if bite site VDK is negative **and** Pt. is envenomed): ______

SNAKE, if available, sent for identification? ☐Yes ☐No If Yes, to whom: ______

ID result: ______

FAX THIS PAGE IMMEDIATELY with a COMPLETED CONSENT FORM to (08) 9224 1489

CLINICAL FEATURES OF ENVENOMING

RESEARCHER OFFICE ONLY
STUDY ID NUMBER: ____

UPDATE THIS SHEET EACH TIME BLOOD IS TAKEN, AND IF SIGNIFICANT CHANGES OCCUR

		ONSET Date/time (24 hr clock)		RESOLVED Date/time (24hr clock)	
**Bite site**					
Pain	Y / N		:		:
Swelling	Y / N		:		:
Bruising	Y / N		:		:
**Regional lymph nodes**					
Pt. aware of pain	Y / N		:		:
Tender on examination	Y / N		:		:
**Non-specific systemic features**					
Nausea	Y / N		:		:
Vomiting	Y / N		:		:
Headache	Y / N		:		:
Abdo pain	Y / N		:		:
Generalised sweating	Y / N		:		:
Diarrhoea	Y / N		:		:
**Coagulopathy / bleeding**					
Bleeding from bite	Y / N		:		:
Bleeding from IV puncture sites	Y / N		:		:
Bleeding from gums	Y / N		:		:
Dipstick urine +ve blood >1+	Y / N		:		:
INTRACRANIAL BLEEDING	Y / N		:		:
GASTROINTESTINAL BLEEDING	Y / N		:		:
Other, specify:			:		:
**Neurotoxicity**					
Ptosis	Y / N		:		:
Poor upgaze / diplopia	Y / N		:		:
Poor lateral gaze / diplopia	Y / N		:		:
Bulbar weakness (cough /gag)	Y / N		:		:
Intercostal weakness	Y / N		:		:
Limb weakness	Y / N		:		:
Reduced FEV1 (record detailed spirometry data in med. record)	Y / N		:		:
**Myotoxicity**					
Muscle pain, bitten limb	Y / N		:		:
Tender muscles, bitten limb	Y / N		:		:
Muscle pain, generalised	Y / N		:		:
Tender muscles, generalised	Y / N		:		:
Trismus / jaw pain	Y / N		:		:
**Cardiovascular**					
Collapse &/or unconscious	Y / N		:		:
**Other** (specify):			:		:
			:		:

MANAGEMENT & BLOOD SAMPLING

RESEARCHER OFFICE ONLY
STUDY ID NUMBER:

REMOVAL OF PRESSURE-IMMOBILISATION

DATE: TIME: : **24 hour clock** PATIENT WEIGHT kg

PREMEDICATIONS (PRIOR TO FIRST DOSE OF ANTIVENOM), IF ANY

MEDICATION	DOSE and ROUTE	TIME	ANY ADVERSE EFFECT?
		:	Yes / No
		:	Yes / No
		:	Yes / No

If YES go to Datasheet 4

ANTIVENOM

TYPE	BATCH NUMBER(S)	No. of vials	Time STARTED	DURATION (Minutes)	ANY ADVERSE EFFECT?
			:		Yes / No
			:		Yes / No
			:		Yes / No
			:		Yes / No

If YES go to Datasheet 4

CLOTTING FACTOR REPLACEMENT (FFP, CRYO etc.)

TYPE	Amount	Time STARTED	DURATION (Minutes)	ANY ADVERSE EFFECT?
		:		Yes / No
		:		Yes / No
		:		Yes / No
		:		Yes / No

If YES go to Datasheet 4

PLEASE RECORD BLOOD SAMPLING TIMES

IMPORTANT NOTE: All cases with coagulopathy must have research bloods at 3 hours post antivenom and a full set of both research bloods and coagulation studies at 6 hours post first dose of antivenom.

DATE	TIMES (24 hour clock)						
	:	:	:	:	:	:	:
	:	:	:	:	:	:	:
	:	:	:	:	:	:	:

LABORATORY RESULTS: **Please attach copies of all investigation results reported by your hospital laboratory.**

ADVERSE REACTION

RESEARCHER OFFICE ONLY
STUDY ID NUMBER:

(You may submit multiple copies if more than one reaction occurs;
either photocopy this datasheet or contact the National Study Line to arrange for another to be faxed)

DATE & TIME OF ONSET **LIKELY CAUSE**

:

dd / mmm / yyyy 24 hour clock

REACTION FEATURES:

Erythema/ Urticaria	Angioedema	Nausea	Vomiting	Abdo/Pelvic Pain	Throat tightness	Chest tightness	Cough
Yes / No	Yes / No	Yes / No	Yes / No	Yes / No	Yes / No	Yes / No	Yes / No

Stridor	Dyspnoea	Wheeze	Accessory Muscle Use	Intercostal indrawing	Hypoxaemia (SpO2<=92%)	Altered consciousness	Diaphoresis
Yes / No	Yes / No	Yes / No	Yes / No	Yes / No	Yes / No	Yes / No	Yes / No

BP Baseline BEFORE Rn	BP LOWEST during Rn	BP HIGHEST during Rn	OTHER:
/	/	/	

EMERGENCY TREATMENT

If space here is insufficient please photocopy and attach drug and fluid administration records

INTERVENTION	DOSE and ROUTE (IF DRUG/FLUID)	TIME
		:
		:
		:
		:

ONCE EMERGENCY TREATMENT HAS BEEN STARTED PLEASE ALSO DO THE FOLLOWING:

1. TAKE ADDITIONAL RESEARCH BLOODS (1xSerum, 1xCitrate) PLUS 1xEDTA (Purple) TUBE and send to the laboratoiry immediately (ON ICE if available)

(i) 10-15 minutes after reaction onset/emergency treatment	Yes / No	TIME TAKEN:	:
(i) One hour after reaction onset/emergency treatment	Yes / No	TIME TAKEN:	:

2. CONTACT THE ASP-FFP INVESTIGATOR to discuss case management and investigation, and to arrange for another Datasheet 4 to be faxed if required.

DATE & TIME OF RESOLUTION OF THE REACTION

:

dd / mmm / yyyy 24 hour clock

NOTES/ COMMENTS:
